# Evidence for lateral gene transfer (LGT) in the evolution of eubacteria-derived small GTPases in plant organelles

**DOI:** 10.3389/fpls.2014.00678

**Published:** 2014-12-11

**Authors:** I. Nengah Suwastika, Masatsugu Denawa, Saki Yomogihara, Chak Han Im, Woo Young Bang, Ryosuke L. Ohniwa, Jeong Dong Bahk, Kunio Takeyasu, Takashi Shiina

**Affiliations:** ^1^Graduate School of Biostudies, Kyoto UniversityKyoto, Japan; ^2^Department of Biology, Faculty of Science, Tadulako UniversityPalu, Indonesia; ^3^Graduate School of Medicine, Kyoto UniversityKyoto, Japan; ^4^Graduate School of Life and Environmental Sciences, Kyoto Prefectural UniversityKyoto, Japan; ^5^Division of Life Science (BK21 plus program), Graduate School of Gyeongsang National UniversityJinju, South Korea; ^6^Division of Biomedical Science, Faculty of Medicine, University of TsukubaTsukuba, Japan

**Keywords:** endosymbiotic gene transfer, genomic analysis, lateral gene transfer, small GTPase, evolution of organelle

## Abstract

The genomes of free-living bacteria frequently exchange genes via lateral gene transfer (LGT), which has played a major role in bacterial evolution. LGT also played a significant role in the acquisition of genes from non-cyanobacterial bacteria to the lineage of “primary” algae and land plants. Small GTPases are widely distributed among prokaryotes and eukaryotes. In this study, we inferred the evolutionary history of organelle-targeted small GTPases in plants. *Arabidopsis thaliana* contains at least one ortholog in seven subfamilies of OBG-HflX-like and TrmE-Era-EngA-YihA-Septin-like GTPase superfamilies (together referred to as Era-like GTPases). Subcellular localization analysis of all Era-like GTPases in Arabidopsis revealed that all 30 eubacteria-related GTPases are localized to chloroplasts and/or mitochondria, whereas archaea-related DRG and NOG1 are localized to the cytoplasm and nucleus, respectively, suggesting that chloroplast- and mitochondrion-localized GTPases are derived from the ancestral cyanobacterium and α-proteobacterium, respectively, through endosymbiotic gene transfer (EGT). However, phylogenetic analyses revealed that plant organelle GTPase evolution is rather complex. Among the eubacterium-related GTPases, only four localized to chloroplasts (including one dual targeting GTPase) and two localized to mitochondria were derived from cyanobacteria and α-proteobacteria, respectively. Three other chloroplast-targeted GTPases were related to α-proteobacterial proteins, rather than to cyanobacterial GTPases. Furthermore, we found that four other GTPases showed neither cyanobacterial nor α-proteobacterial affiliation. Instead, these GTPases were closely related to clades from other eubacteria, such as *Bacteroides* (Era1, EngB-1, and EngB-2) and green non-sulfur bacteria (HflX). This study thus provides novel evidence that LGT significantly contributed to the evolution of organelle-targeted Era-like GTPases in plants.

## Introduction

Plant cells contain two types of endosymbiotic organelle, chloroplasts and mitochondria, which arose from cyanobacterium and α-proteobacterium-like ancestors, respectively. During the course of plant evolution, many cyanobacterium and α-proteobacterium-derived genes were either lost from the organelles or transferred to the nucleus (endosymbiotic gene transfer: EGT). Thus, extant chloroplasts and mitochondria retain many prokaryotic proteins that are encoded by the nuclear genome, whereas organelle genomes encode a limited number of proteins.

Lateral gene transfer (LGT) refers to the transmission of genetic material between distinct evolutionary lineages, and plays a substantial role in generating the diversity of genes in host cells. It is well known that LGT is an important process in the evolution of prokaryotes, particularly in the evolution of antibiotic resistance (Barlow, 2009). In contrast to prokaryotic cells, LGT between multicellular eukaryotes is generally believed to be rare, due to the barrier of germline in multicellular animals and apical meristem in plants (Andersson, 2005; Bock, 2010). However, several lines of evidence suggest that there were ancient gene transfers from non-cyanobacterial bacteria to the lineage of “primary” algae and land plants. For example, *Arabidopsis thaliana* has 24 genes of chlamydial origin (Qiu et al., 2013). Furthermore, at least 55 Chlamydiae-derived genes have been identified in algae and plants, most of which are predominantly involved in plastid functions (Moustafa et al., 2008), suggesting an ancient LGT from Chlamydiae to the ancestor of primary photosynthetic eukaryotes (Huang and Gogarten, 2007; Becker et al., 2008; Moustafa et al., 2008; Ball et al., 2013). Moreover, extensive analysis of plastid proteome data revealed that 15% of Arabidopsis plastid proteins are originated through HGT from non-cyanobacterial bacteria, including Proteobacteria and Chlamydiae (Qiu et al., 2013). In addition, five shikimate pathway proteins in chloroplasts have also been obtained by LGT from β/γ-proteobacteria and *Rhodopirellula baltica* (Richards et al., 2006). It is known that some secondary plastid-containing unicellular algae acquired many chloroplast-targeted proteins through LGT from non-cyanobacterial bacteria (Archibald et al., 2003; Nosenko et al., 2006; Grauvogela and Petersen, 2007; Teicha et al., 2007). Furthermore, recent genome analysis of the moss *Physcomitrella patens* provided evidence for the impact of LGT on the acquisition of genes involved in several plant specific processes during the evolution of early land plants (Yue et al., 2012). These results suggest that LGT plays a more important role in the evolution of plants than previously thought.

**Figure 1:**
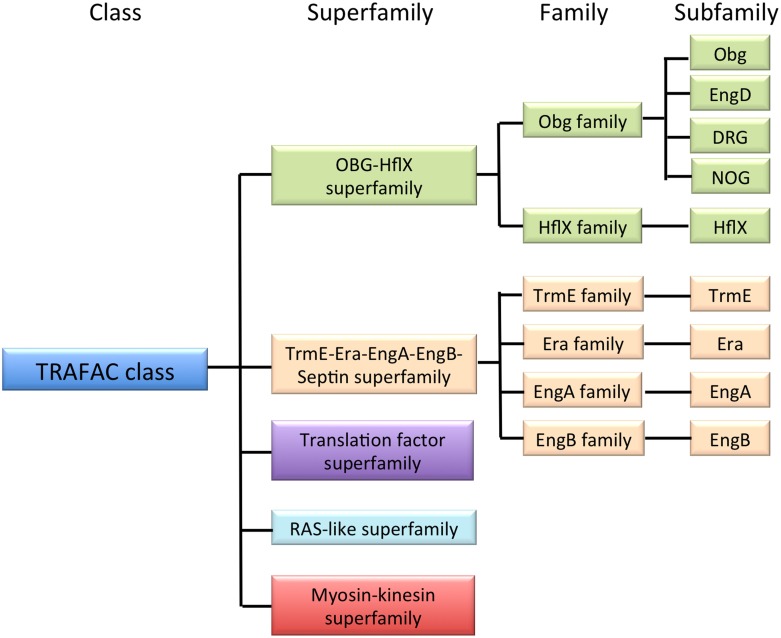
**Classification of GTPases**. The TRAFAC class is a member of the P-loop GTPase superclass and is composed of conserved protein superfamilies, as shown. The OBG-HflX-like superfamily and the TrmE-Era-EngA-YihA-septin-like superfamily together contain nine subfamilies (Era-like GTPases).

The small GTP-binding proteins (GTPases) are found in all domains of life. They are critical regulators of many aspects of basic cellular processes, including translation, cellular transport and signal transduction. Comprehensive genome sequence analysis has revealed that the TRAFAC (translation factor) class GTPases can be divided into five superfamilies, among which are the OBG-HflX-like and TrmE-Era-EngA-YihA-Septin-like superfamilies (Figure 1). The OBG-HflX superfamily consists of the Obg and HflX families, and the Obg family can be further divided into four subfamilies: Obg, EngD, Drg, and Nog1 (Leipe et al., 2002; Verstraeten et al., 2011). The TrmE-Era-EngA-YihA-Septin superfamily is made up of the TrmE, Era, EngA, EngB families. The OBG-HflX-like and TrmE-Era-EngA-YihA-Septin-like superfamilies (hereafter, together referred to as Era-like GTPases) are represented by *Obg* and *Era*, which were identified originally in *Bacillus subtilis* and *Escherichia coli*, respectively. Obg proteins are involved in multiple cellular processes, including cell growth (Morimoto et al., 2002), morphological differentiation, DNA replication (Slominska et al., 2002), chromosome partitioning (Kobayashi et al., 2001) and the regulation of protein synthesis and/or ribosome functions (Datta et al., 2004; Sato et al., 2005; Schaefer et al., 2006) in *Bacillus subtilis* and other eubacteria. Era has also been shown to play an important role in the cell cycle and ribosome assembly (Britton et al., 1998) by binding to 16S rRNA in *E. coli* (Hang and Zhao, 2003) and to the 30S ribosomal subunit in *E. coli* and *B. subtilis* (Morimoto et al., 2002). Other Era-like GTPases are also known to be involved in ribosome maturation and/or RNA modification in eubacteria.

Among the subfamilies composing the Era-like GTPases, seven are eubacterium-related (Obg, HflX, TrmE, EngD, EngB, Era, and EngA) and conserved from eubacteria to eukaryotes, whereas two are archaea-related (Nog1 and Drg) and conserved in eukaryotes. It is expected that the eubacterium-related GTPase genes were acquired through EGT in eukaryotic cells and are localized to the symbiotic organelles, namely mitochondria and chloroplasts. On the other hand, the archaea-related DRG and NOG1 must have originated from an archaeaic host cell, and likely function in cytoplasm and/or nuclei. However, subcellular localization and functions of the Era-like GTPases remain largely unknown in eukaryotes, except for Obg, Drg, and Nog1. It has been shown that Obg homologs are targeted to mitochondria in yeast (Datta et al., 2005), and to mitochondria and the nucleolus in human cells (Hirano et al., 2006). By contrast, Drg and Nog1 GTPases play important roles in the cytoplasm and mitochondria, respectively, in animal and yeast cells (Mittenhuber, 2001; Park et al., 2001). These results suggest that the Era-like GTPases may be involved in the regulation of organelle functions in eukaryotes.

Genomic data on the Era-like GTPase genes show that plants have a larger number of GTPase genes than do bacteria, yeast, or mammals (Leipe et al., 2002). It is expected that plants acquired additional chloroplast-localized GTPases from cyanobacteria through EGT (McFaddan, 2001). In fact, Bang et al. (2009, 2012) reported that there are two Obg homologs that target to chloroplasts and mitochondria in Arabidopsis. However, very little is known about the intracellular compartmentation and evolution of other Era-like GTPases in plants. To address these questions, we performed comprehensive phylogenetic and subcellular localization analyses of eubacterial Era-like GTPase proteins in Arabidopsis. We found that all 13 eubacteria-related GTPases (of the Obg, HflX, TrmE, EngD, EngB, Era, and EngA subfamilies) were localized to chloroplasts and/or mitochondria in Arabidopsis, whereas archaea-related DRG and NOG1 were localized to the cytoplasm and nuclei, respectively. Unexpectedly, however, EGT likely played a limited role in the evolution of chloroplast and mitochondrial GTPases. There were only three chloroplast GTPases and one dual-targeting GTPase derived from the ancestral cyanobacterium and two mitochondrial GTPases derived from the ancestral α-proteobacterium through EGT. On the other hand, three chloroplast other GTPases were related to α-proteobacterial proteins, but not to cyanobacterial GTPases, suggesting re-compartmentation of mitochondrial GTPases to chloroplasts during plant evolution. Moreover, four Era-like GTPases were closely related to clades from other eubacteria, such as Bacteroides (Era1, EngB-1, and EngB-2) and green non-sulfur bacteria (HflX). These results suggest that LGT from Bacteroides and green non-sulfur bacteria has played a significant role in the evolution of genes for chloroplast- and mitochondria-target GTPases in land plants.

## Materials and methods

### Phylogenetic analyses and classification

Obg/Era superfamily genes were retrieved from public databases (NCBI, TAIR, and KEGG) by genome screening with the known amino acid sequences of members of each subfamily as queries. Genes that are only detected in the query and potential donor groups will also be identified. Detailed phylogenetic analyses were performed for each of the candidates. Taxonomic distribution of sequence homologs was also investigated.

Multiple protein sequence alignments were performed using the Clustal X program (Jeanmougin et al., 1998) followed by manual refinement. Gaps and ambiguously aligned sites were removed manually. The well-aligned regions were used for the construction of phylogenetic trees. Phylogenetic analyses were performed using the protdist program with JTT amino acids substitution model, and followed by neighbor program in the PHYLIP 3.6 package (Ratief, 2000). The phylogenetic tree was inferred using the neighbor-joining method (Saitou and Nei, 1987) and tested using 100 replications of bootstrap analysis using the seqboot and consense programs in the same package. The data were subsequently visualized as phylogenetic trees using the treeview program (Page, 1996). The names and classifications proposed herein are based on P-loop protein classification (Leipe et al., 2002).

### Plant and cell growth conditions

*Arabidopsis thaliana* ecotype Colombia were germinated and grown on Murashige–Skoog (MS) medium containing 0.8% (w/v) agar and 1% (w/v) sucrose at 22°C with 80–100 μmol m^−2^ s^−1^ illumination for a daily 16-h light period. Arabidopsis suspension-culture cells were cultured in MS medium at 23°C with continuous agitation under dark conditions. Onion bulb was purchased from local market.

### Molecular cloning and transient expression assays

GFP fusion genes were constructed as follows. First-strand cDNA was synthesized from total RNA prepared from Arabidopsis seedlings using AMV reverse transcriptase (TaKaRa). cDNA was amplified by PCR using *KOD-plus*-DNA polymerase (TOYOBO) according to the manufacturer's protocol. Transient expression vectors were constructed using the *GFP* reporter plasmid 35Ω-sGFP(S65T). The PCR fragments containing full length *Era*-like GTPase genes were ligated in frame into the 35Ω-sGFP(S65T) plasmid. All sets of primers used in this study are listed in Supplemental data 1. Transient expression of the GFP fusion proteins in Arabidopsis protoplasts was performed as previously described (Yanagisawa et al., 2003). Briefly, rosette leaves of 4–6-week-old plants were used for the transient expression experiments. After overnight incubation at 23°C in the dark, GFP signal was observed using a confocal laser scanning microscope (LSM5 PASCAL; Carl Zeiss Inc.) equipped with green HeNe and argon lasers. The assay using Arabidopsis culture cells was performed as previously described (Uemura et al., 2004). Mitochondrial GTPases were transiently expressed in onion epidermal cells by using particle bombardment. 1.5 μg of GFP fusion plasmids coated on 0.6 μm gold particles were bombarded into epidermal sheaths peeled from onion bulbs placed on ½ MS plates. The epidermal cells were stained with MitoTracker Red to label the mitochondria. Expression assays were performed with at least three independent repetitions and mitochondrial signals were confirmed by MitoTracker Red staining.

### Isolation of mitochondria from arabidopsis seedlings and immunoblot analysis

Intact mitochondria were isolated from Arabidopsis hydroponic seedling cultures as described previously (Sweetlove et al., 2007). Mitochondria were subsequently separated into membrane and soluble fractions. Immunoblot analyses of the mitochondrial fractions were performed using antibodies against *E. coli* ObgE and mitochondrial outer membrane marker, voltage-dependent anion-selective channel protein (VDAC).

## Results

### Era-like GTPase proteins in plants

We conducted genome-wide searches for proteins containing Era-like GTPase signatures to identify all Era-like GTPases in three model plant genomes: *Arabidopsis thaliana* (dicot), *Oryza sativa* (monocot), and *Cyanidioschyzon merolae* (red algae). Arabidopsis was found to have 18 GTPase genes, including members of all nine Era-like GTPase subfamilies (Table 1). Arabidopsis, rice and *C. merolae* had at least one gene in each of the nine subfamilies, suggesting that plants require similar sets of *Obg*/*Era* GTPase genes. Furthermore, humans have the same sets of genes as plants, except for *EngA*, suggesting that Obg, Drg, NOG1, EngD, HflX, TrmE, Era, and EngB subfamily genes are shared between plants and animals. By contrast, *S. cerevisiae* lacks *HflX*, *Era*, and *EngA* genes, suggesting that the unicellular fungi Saccharomyces has lost several gene sets during evolution. *Drg* and *Nog1* belong to the Obg family, and were found in two domains of life, archaea and eukaryotes, but not in eubacteria (Suwastika et al., 2014). By contrast, *Obg*, *EngD*, *HflX*, *TrmE*, *Era*, *EngA*, and *EngB* genes were found in eubacteria and eukaryotes (Table 1). It is likely that the archaea-related genes were derived from a eukaryotic host cell, but eubacteria-related genes from eubacterial ancestors. Both *HflX* and *EngB* are also shared among eubacteria, eukaryotes and some archaea.

Table 1**OBG-Hflx-like Superfamily and TrmE-Era-EngA-YihA-Septin-like Superfamily genes in Arabidopsis genom**.

**Table:** 

**OBG-HflX-like SUPERFAMILY**								
**Fam**.	**Sub fam**.	***A. thaliana***	**Name**	***O. zativa***	***C. merolae***	***S. sereviciae***	***H. sapien***	***E.coli***
Obg	Obg	At1g07615	Obg A-1	Os03g58540	CMG146C	YHR168W	hsa26164	JW3150
		At5g18570	Obg A-2	Os07g47300			hsa85865	
				Os11g47800				
	EngD/YyaF/YchF	At1g30580	EngD-1	Os08g019930	CME188C	YRR025C	hsa29789	JW1194
		At1g56050	EngD-2		CMT184C	YHL014C		
	Ygr210					YGR210C		
	Drg	At4g39520	Drg1-1	Os07g43470	CMG124C	YAL036C	hsa151457	
		At1g17470	Drg1-2	Os05g28940	CMN324C	YGR173W	hsa1819	
		At1g72660	Drg1-3				hsa4733	
	Nog	At1g50920	Nog1-1	Os07g01920	CMB146C	YPL093W	hsa23560	
		At1g10300	Nog1-2	Os06g09570				
Hflx		At5g57960	Hflx	Os03g51820	CMT373C		hsa8225	JW4131
				Os11g38020

**Table:** 

**TrmE-Era-EngA-YihA-Septin-like SUPERFAMILY**							
**Fam**.	***A. thaliana***	**Name**	***O. zativa***	***C. merolae***	***S. sereviciae***	***H. sapien***	***E. coli***
TrmE/ThdF	At1g78010	TrmE	Os08g31460	CMK223C	YMR023C	hsa84705	JW3684
				CMV025C			
FeoB							JW3372
EngB/YihA	At2g22870	EngB-1	Os03g23250	CMQ232C	YDR336W	hsa29083	JW5930
	At5g11480	EngB-2	Os01g73220				
	At5g58370	EngB-3	Os03g81640				
Era	At5g66470	Era-1	Os05g49220	CMN201C		hsa26284	JW2550
	At1g30960	Era-2					
EngA/YfgK	At3g12080	EngA-1	Os01g12540	CMC059C			JW5403
	At5g39960	EngA-2	Os11g41910				

**Figure 2:**
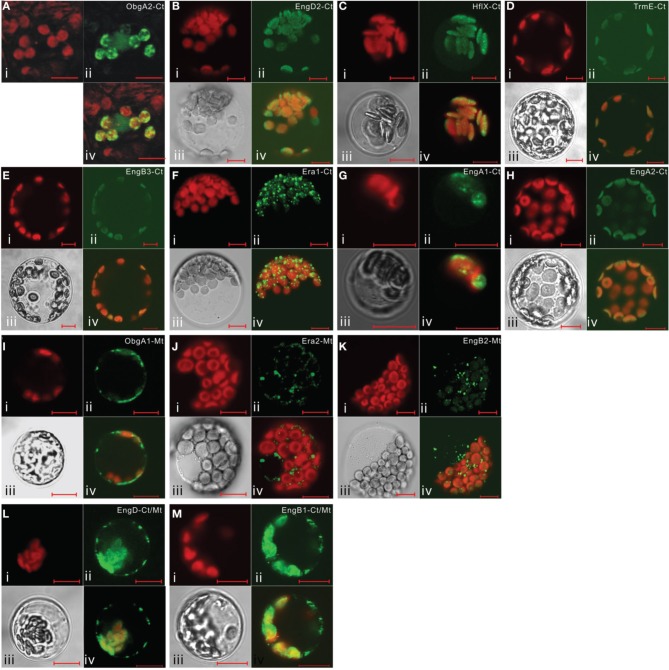
**Subcellular localization of eubacterium-related Era-like GTPases in Arabidopsis**. Transient expression of GFP-fusion proteins in Arabidopsis protoplasts: **(A–H)** chloroplast targeting of ObgA2, EngD2, Hflx, TrmE, EngB3, Era1, EngA1, and EngA2 proteins. **(I–K)** mitochondrial targeting of ObgA1, Era2, and EngB2 proteins. **(L,M)** dual targeting of EngD1 and EngB1 to mitochondria and chloroplasts. (i) Chlorophyll auto-fluorescence, (ii) GFP fluorescence, (iii) DIC image, (iv) merged image of (i), (ii), and (iii). Scale bars are 10 μm.

**Figure 3:**
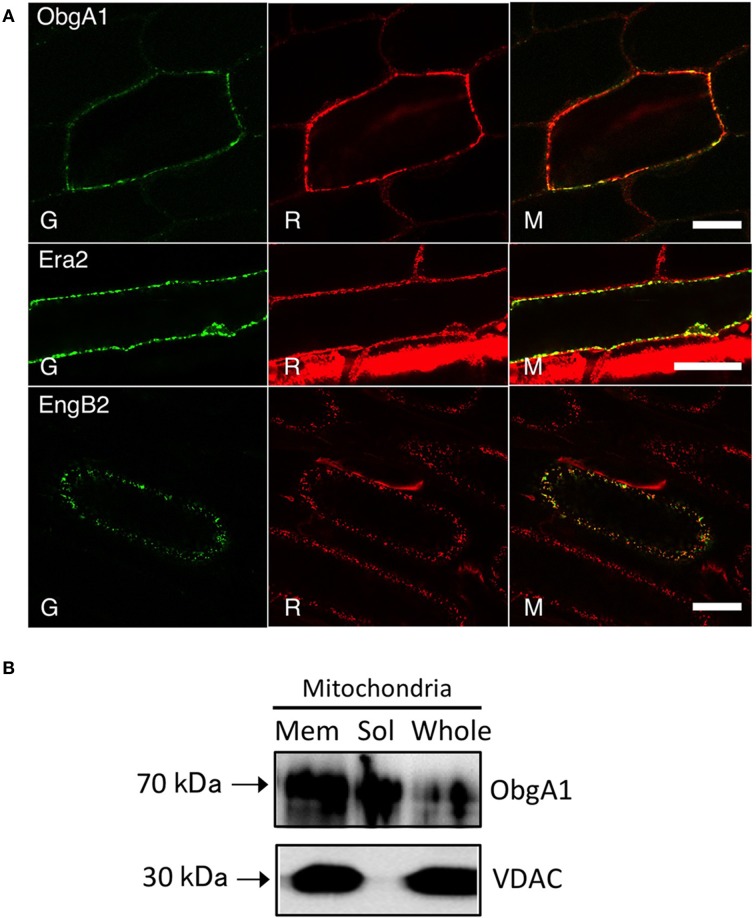
**Mitochondrial localization of ObgA1, Era2, and EngB2 proteins**. **(A)** Confocal images of ObgA1-GFP, Era2-GFP, and EngB2-GFP fusion proteins transiently expressed in onion epidermal cells. All proteins were targeted to mitochondria as confirmed by mitotracker staining. G, GFP fluorescence; R, mitotracker Red; M, merged image. Scale bars are 10 μm. **(B)** Western blot analysis of ObgA1 in mitochondria fractions. The Arabidopsis mitochondria whole lysates (whole) were fractionated into membrane (Mem) and matrix (Sol) fractions. Fractions were resolved on a 10% SDS-PAGE and detected with the anti-ObgE and anti-VDAC (mitochondrial outer membrane marker) antibodies. Ten micrograms protein were loaded.

**Figure 4:**
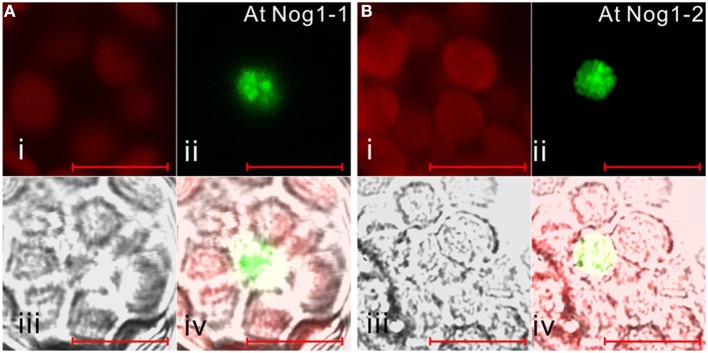
**Subcellular localization of archaea-related Nog1 in Arabidopsis**. GFP-fusion Nog1-1 **(A)** and Nog1-2 **(B)** proteins are transiently expressed in Arabidopsis protoplasts. (i) Chlorophyll auto-fluorescence, (ii) GFP fluorescence, (iii) DIC image, (iv) merged image of (i), (ii), and (iii). Scale bars are 10 μm.

It is noteworthy that vacsular plants have a larger number of Era-like GTPase genes (18 genes in Arabidopsis and 17 genes in rice) compared to human (11 genes) and yeast (9 genes) (Table 1). Although the human genome contains a single gene of each Era-like GTPase subfamily except for the Obg and Drg subfamilies, plant Era-like GTPase subfamilies contain multiple genes. It is predicted that multiple Era-like GTPase proteins are targeted to different cellular compartments, such as chloroplasts, mitochondria and nuclei (Table 2). However, the subcellular localization of most Obg/Era superfamily proteins has not been determined in plants, except for chloroplastic and mitochondrial Obg proteins (Bang et al., 2009). In this study, we examined subcellular localization of Era-like GTPases in Arabidopsis using *in vivo* analysis of GFP-tagged proteins. C-terminal GFP fusions were transiently expressed in Arabidopsis protoplasts or cultured cells under the transcriptional control of the cauliflower mosaic virus 35S promoter. As predicted, all eubacterium-related GTPases were localized in chloroplasts and/or mitochondria, but not other organelles nor cytoplasm (Figure 2). We identified eight proteins that were targeted exclusively to chloroplasts (Figures 2A–H) and two dual-targeting proteins transported into both chloroplasts and mitochondria (Figures 2L,M). Interestingly, each family/subfamily contained at least one chloroplast protein, suggesting that eubacteria-related Era-like GTPases play an important role in chloroplasts (Table 2). On the other hand, only three mitochondrion-specific proteins (ObgA1, Era2 and EngB2) were identified (Figures 2I–K). The colocalization of the GFP fluorescence with the red fluorescence of the MitoTracker dye confirms the mitochondrial targeting of these respective GFP fusions in onion epidermal cells (Figure 3A). Mitochondrial localization of ObgA1 was further confirmed by western blotting analysis of mitochondrial fractions isolated from Arabidopsis seedlings. Anti-ObgE antibody specifically detected ObgA1 in both membrane and soluble fractions of mitochondria (Figure 3B). By contrast, all Drg GTPases were localized to the cytoplasm in Arabidopsis (Suwastika et al., 2014), whereas NOG1 homologs were localized to the nucleus (Figure 4).

**Table 2:** **Subcellular localization of Obg-TrmE GTPases in Arabidobsis**.

**Subfamily**	**Gene name**	**AGI number**	^*^TargetP/Wolf PSORT	**Proteome**	^**^GFP
Obg	Obg A1	At1g07615	Mit 6/Chl 4, Mit 4	n.d	Mit
Subfamily	Obg A2	At5g18570	Mit 9/Chl 8	Chl	Chl
EngD	EngD-1	At1g30580	Other 5/Cysk 8	Cyto	Chl/Mit
	EngD-2	At1g56050	Chl 3/Chl 10	Chl	Chl
Drg^***^	Drg1-1	At4g39520	Other 8/Cyto 7	Cyto/PM	Cyto
	Drg1-2	At1g17470	Other 8/Cyto 7	n.d	Cyto
	Drg1-3	At1g72660	Other 8/Cyto 7	n.d	Cyto/Nucl
Nog	Nog1-1	At1g50920	Other 8/Cyto 8	PM	Nucl
	Nog1-2	At1g10300	Other 8/Chl 6	n.d	Nucl
Hflx	Hflx	At5g57960	Chl 8/Chl 7	Chl	Chl
TrmE	TrmE	At1g78010	Chl 5, Mit 3/Chl 9	Chl	Chl
Era	Era 1	At5g66470	Chl 8/Mit, Chl 3	Chl	Chl
	Era 2	At1g30960	Mit 9/Chl 5, Mit 5	n.d	Mit
EngA	EngA-1	At3g12080	Chl 8/Mit 5, Chl 4	Chl	Chl
	EngA-2	At5g39960	Mit 7/Chl 6, Mit 3	n.d	Chl
EngB	EngB-1	At2g22870	Mit 7/Chl 7	n.d	Chl/Mit
	EngB-2	At5g11480	Chl 7/Chl 8	n.d	Chl/Mit
	EngB-3	At5g58370	Mit 5/Chl 4, nucl 3	n.d	Chl

* *Prediction*.

** *Results of this expreriment*.

*** *Suwastika et al. (2014)*.

*n.d., no data*.

### Chloroplast-targeted Obg and TrmE are of cyanobacterial origin

**Figure 5:**
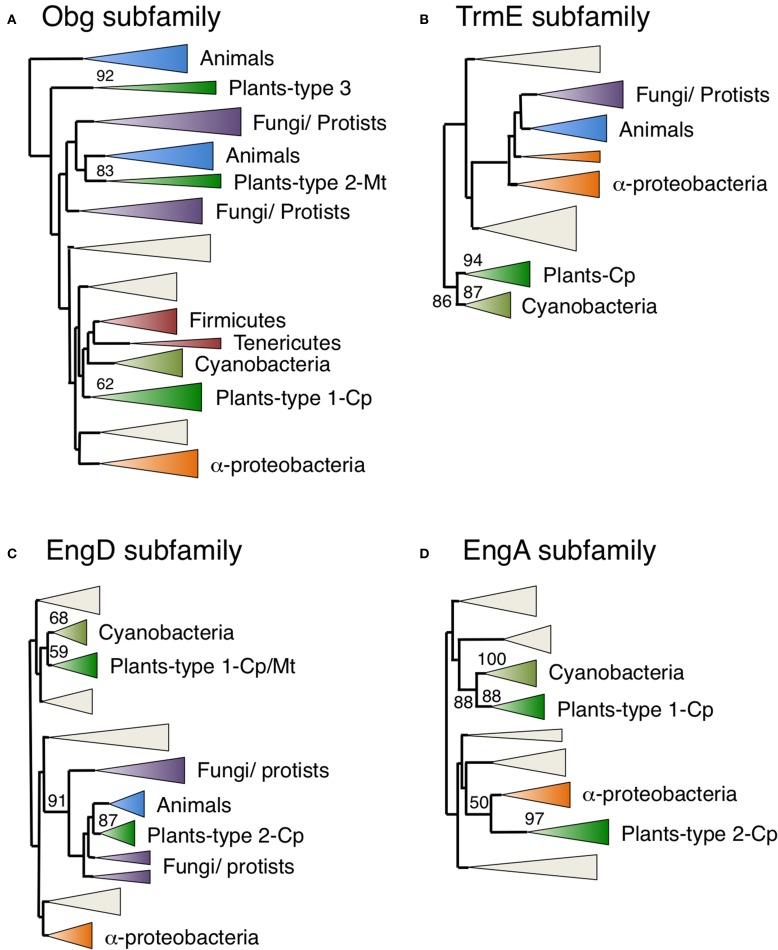
**Phylogenetic tree of Obg, TrmE, EngD, and EngA subfamily proteins**. Comprehensive comparison of Obg **(A)**, TrmE **(B)**, EngD **(C)**, and EngA **(D)** subfamily proteins in eukaryotes, eubacteria and archaea. Sequences were aligned using Clustal X based on 185 (Obg), 152 (TrmE), 182 (EngD), and 147 (EngA) proteins. The tree was inferred using the neighbor-joining method with JTT model. Numbers at the nodes indicate bootstrap values obtained for 100 replicates. The horizontal length of the triangles is equivalent to the average branch length. Green triangles, plant clade; light green triangles, cyanobacterial clade; blue triangles, animal clade; purple triangles, fungus-protist clade; orange triangles, α-proteobacteria clade. The original phylogenetic trees are shown in Figures S1–S4.

*Obg* and *TrmE* genes are found in eubacteria, animals, fungi and plants (Table 1). Several lines of evidence imply that Obg GTPases function in ribosome maturation in eubacteria (Sato et al., 2005), mitochondria of yeast (Datta et al., 2005) and human nuclei (Hirano et al., 2006). Figure 5, Figure S1 portray a NJ tree of Obg homologs, demonstrating that plant Obg homologs formed three distinct monophyletic clusters (types 1–3) with robust support of 62, 83, and 92%, respectively. Arabidopsis had two Obg homologs, ObgA1 (At1g07615) and ObgA2 (At5g18570). ObgA2 (ObgC/Obg target to chloroplast) in the type 1 cluster has been shown to be localized to chloroplasts (Bang et al., 2009; Figure 2A). GFP-tagged ObgA2 appeared in small dot-like structures in chloroplasts, suggesting that ObgA2 is associated with chloroplast nucleoids. The type 1 plant Obg homologs were closely related to cyanobacterial homologs, suggesting that they have cyanobacterial endosymbiotic ancestry.

By contrast, the type 2 plant Obg homologs were closely related to animal and fungal homologs. The human Obg homolog ObgH1 is localized to mitochondria in HeLa cells (Hirano et al., 2006). Similarly, we showed that Arabidopsis ObgA1 (a type 2 Obg) was also exclusively localized in mitochondria (Figures 2I, 3A,B). However, it should be noted that there was not a close relationship between type 2 plant Obg and α-proteobacterial Obg. The chloroplast and cyanobacterium-like Obg proteins have a TGS domain in the C-terminal region, whereas mitochondrial Obg proteins lack the TGS domain. The TGS domain is known to be involved in stress responses in eubacteria. Therefore, chloroplast Obg GTPases might have specific a role in plant stress responses.

The type 3 plant Obg proteins were related to another animal Obg homologs, represented by ObgH2, which is localized in nucleus (Hirano et al., 2006). Plants including green algae, moss and some vacsular plants have one type 3 Obg homolog, whereas Arabidopsis lacks the type 3 Obg. The subcellular localization of type 3 plant Obg homologs remains to be examined.

Finally, it is noteworthy that *C. merolae* retained the Type 1 chloroplast Obg homolog, but lacked the type 2 and type 3 mitochondrial and nuclear Obg homologs. It is conceivable that type 1 Obg or other Obg-related proteins might take over the function of mitochondria Obg in *C. merolae*.

On the other hand, green plant TrmE proteins formed a single monophyletic group that was closely related to a cyanobacterial clade with a strong bootstrap value (87%) (Figure 5, Figure S2), supporting their cyanobacterial endosymbiotic ancestry. In fact, Arabidopsis TrmE protein was targeted exclusively to chloroplasts. *E. coli* TrmE is involved in the modification of uridine bases at the first anticodon of tRNA. Therefore, plant TrmE might have a role in tRNA modification in chloroplasts. It should be noted that animal and fungal proteins form distinct clades that are unrelated to plant proteins, but are grouped with α-proteobacterial genes. The TrmE protein is known to be targeted to mitochondria in yeast (Decoster et al., 1993; Colby et al., 1998), suggesting that mitochondrial TrmE was derived from α-proteobacteria. Interestingly *C. merolae* has two animal-related *TrmE* genes but not the cyanobacterium-related chloroplast genes. It is likely that *C. merolae* has lost the cyanobacterium-derived TrmE gene, while green plants have lost the animal-type mitochondrial *TrmE* during evolution. It is possible that other mitochondrion-localized GTPases have taken over the function of mitochondrial TrmE in green plants.

### Chloroplast-targeted EngD and EngA are of α-proteobacterial origin

*EngD* and *EngA* encode GTP-dependent nucleic acid binding protein (Tomar et al., 2011) and 50S ribosome associated protein (Bharat et al., 2006), respectively. Both plant EngD and EngA homologs formed two monophyletic clusters. The type 1 plant clusters grouped with cyanobacterial clusters with 68% support for EngD1 (Figure 5, Figure S3) and 88% for EngA1 (Figure 5, Figure S4). On the other hand, the type 2 EngD2 and EngA2 proteins formed monophyletic clusters with 87 and 97% support, respectively, and were closely related to animal/fungal and/or α-proteobacterial genes. *C. merolae* also had two EngD proteins that were divided into type 1 and type 2 groups, and one EngA related to the type 1 group. These results suggest that type 1 EngD and EngA proteins were derived from cyanobacterial endosymbiotic ancestors, whereas type 2 EngA proteins were derived from the α-proteobacterial endosymbiont via EGT. The type 1 cyanobacterium-related EngD1 was localized in both chloroplasts and mitochondria (dual targeting; Figure 2L), whereas EngA1 was localized exclusively to chloroplasts (Figure 2G). Interestingly, the type 2 α-proteobacteria-related EngD2 (Figure 2B) and EngA2 GTPases (Figure 2H) were also exclusively targeted to chloroplasts. These findings support the idea that chloroplasts acquired additional type 2 EngD2 and EngA2 GTPases through re-compartmentation of α-proteobacterium-related GTPases from mitochondria.

### Chloroplast-localized HflX might be derived from green non-sulfur bacteria through lateral gene transfer

**Figure 6:**
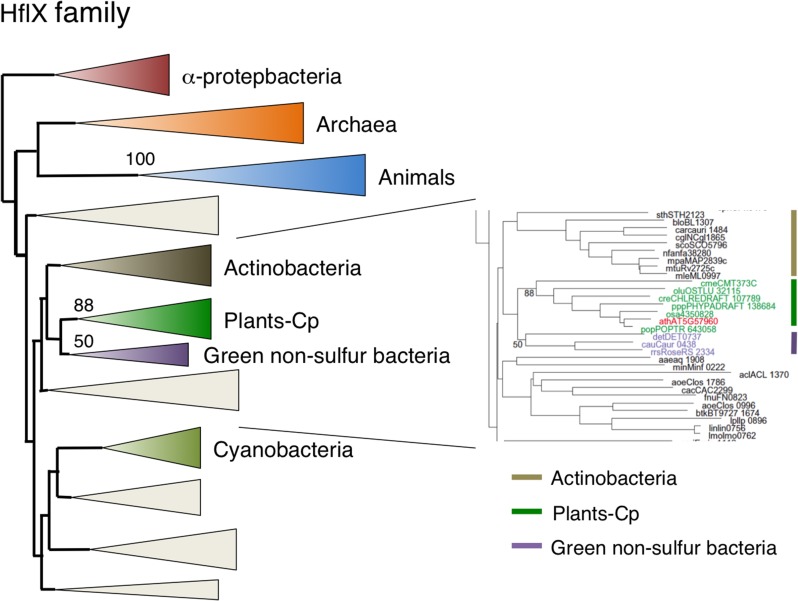
**Phylogenetic tree of HflX subfamily proteins**. Comprehensive comparison of HflX subfamily proteins in eukaryotes, eubacteria and archaea. Sequences were aligned using Clustal X based on 153 proteins. The tree was inferred using the neighbor-joining method with JTT model. Numbers at the nodes indicate bootstrap values obtained for 100 replicates. The horizontal length of the triangles is equivalent to the average branch length. Green triangle, plant clade; light green triangle, cyanobacterial clade; blue triangle, animal clade; orange triangle, α-proteobacteria clade; dark blue triangle, archaea clade; yellow triangle, green non-sulfur clade. The original phylogenetic tree is shown in Figure S5.

*HflX* genes are widely conserved among eubacteria, eukaryotes, and some archaea. It was demonstrated recently that Chlamydophila HflX is associated with the 50S ribosome, suggesting a possible role in ribosome maturation and translational regulation (Polkinghorne et al., 2008). Animal HflX homologs formed a monophyletic group with 100% bootstrap support, and were closely related to the archaeal clade (Figure 6, Figure S5), suggesting that animal HflX genes were derived from archaeal ancestors. By contrast, plants lack archaea-like genes. Arabidopsis had a single HflX homolog that was exclusively localized in chloroplasts (Figure 2C). Phylogenetic analysis revealed that plant HflX homologs form a single monophyletic group with strong bootstrap support (88%). Unexpectedly, however, the plant HflX clade was not related to the cyanobacterial or animal clades, but instead was closely related to the green non-sulfur bacteria group. It is conceivable that the plant HflX genes were derived from green non-sulfur bacteria through LGT. The plant clade included the protein from the primitive red algae *C. merolae*, suggesting that the gene transfer occurred at a very early stage in plant evolution before the red algae lineage and green plant lineage diverged.

### Chloroplast-localized Era1 is derived from green sulfur bacteria or bacteriodes, but not cyanobacteria

**Figure 7:**
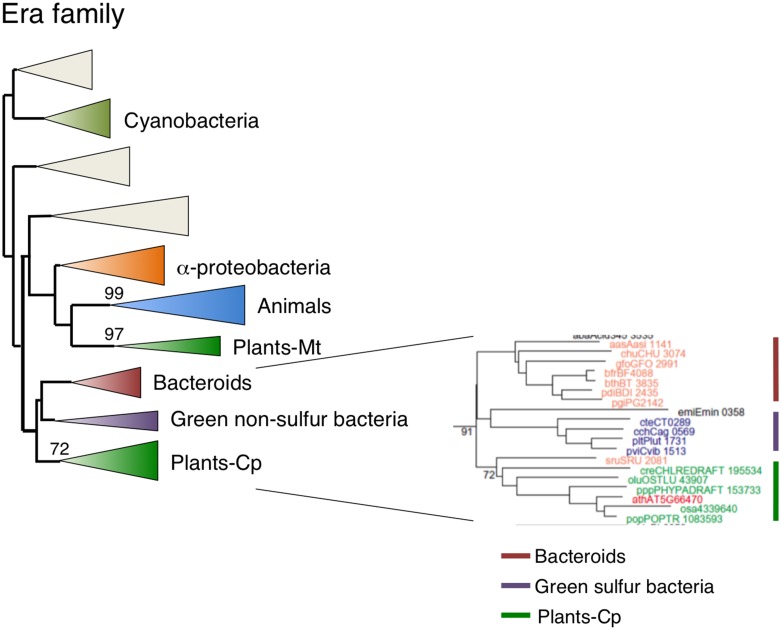
**Phylogenetic tree of Era subfamily proteins**. Comprehensive comparison of Era subfamily proteins in eukaryotes, eubacteria and archaea. Sequences were aligned using Clustal X based on 141 genes. The tree was inferred using the neighbor-joining method with JTT model. Numbers at the nodes indicate bootstrap values obtained for 100 replicates. The horizontal length of the triangles is equivalent to the average branch length. Green triangle, plant clade; light green triangle, cyanobacterial clade; blue triangle, animal clade; orange triangle, α-proteobacteria clade; yellow triangle, green non-sulfur clade; red triangle, bacteroides clade. The original phylogenetic tree is shown in Figure S6.

As a homolog of RAS, Era is an extremely important GTPase in *E. coli*. It has been suggested that Era is directly associated with the 30S ribosomal subunits (Sayed et al., 1999). Human Era (ERAL1) is involved in the regulation of apoptosis (Akiyama et al., 2001). Arabidopsis had two Era homologs: type 1 Era-1 was targeted to chloroplasts (Figure 2F) and type 2 Era2 was a mitochondrial protein (Figures 2J, 3A). GFP-tagged Era1 appeared in small dot-like structures that were observed throughout chloroplasts, suggesting that Era1 is associated with chloroplast nucleoids. Plant Era2 homologs formed a monophyletic group with robust support of 97% and grouped with clusters of animal and α-proteobacteria (Figure 7, Figure S6), suggesting that mitochondrial Era genes were derived from the symbiotic α-proteobacterial ancestors. By contrast, type 1 Era homologs formed a distinct monophyletic group (91%) with Bacteriodes and Green sulfur bacteria clusters. In particular, *Salinibacter rubber* (Bacteroidetes) was placed at the base of the plant lineage. Cyanobacterial Era homologs formed a separate monophyletic group and were not related to either type 1 or type 2 plant Era clusters. This lineage-specific bacterial affiliation of chloroplast-targeted Era implies that there was LGT from Bacteriodes/Green sulfur bacteria to the plant ancestor. Type 2 mitochondrial Era was conserved in the primitive red alga *C. merolae*, but the type 1 chloroplast Era was not.

### Dual-targeting EngB is derived from bacteroides via lateral gene transfer

**Figure 8:**
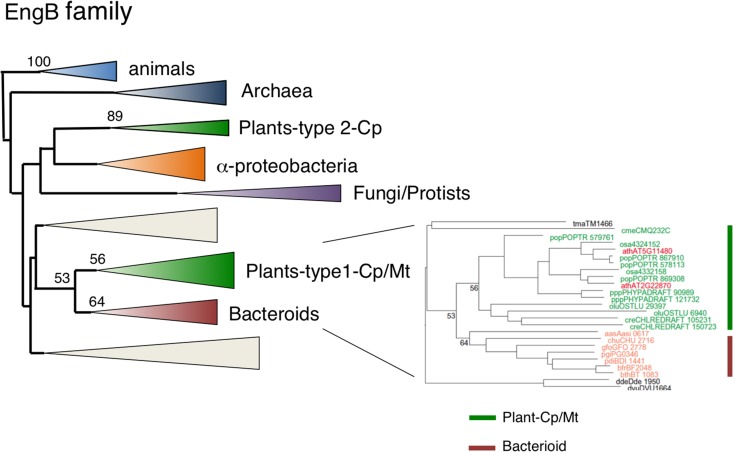
**Phylogenetic tree of EngB family proteins**. Comprehensive comparison of EngB subfamily proteins in eukaryotes, eubacteria and archaea. Sequences were aligned using Clustal X based on 143 proteins. The tree was inferred using the neighbor-joining method with JTT model. Numbers at the nodes indicate bootstrap values obtained for 100 replicates. The horizontal length of the triangles is equivalent to the average branch length. Green triangle, plant clade; light green triangle, cyanobacterial clade; blue triangle, animal clade; purple triangle, fungus-protist clade; orange triangle, α-proteobacteria clade; yellow triangle, green non-sulfur clade; red triangle, Bacteroides clade. The original phylogenetic tree is shown in Figure S7.

*EngB* (*YihA*) has been characterized as an essential gene of unknown function in both *E. coli* and *B. subtilis* (Arigoni et al., 1998; Dassain et al., 1999). Arabidopsis encodes three EngB proteins: EngB1 was dual targeted to chloroplasts and mitochondria (Figure 2M), whereas EngB3 was localized exclusively in chloroplasts (Figure 2E). By contrast, EngB2 was localized exclusively to mitochondria (Figures 2K, 3A). Phylogenetic analysis revealed that plant EngB proteins formed two distinct monophyletic clusters: type 1 and type 2 clusters with 56 and 89% support, respectively (Figure 8, Figure S7). The type 1 cluster, including dual-targeting EngB1 and mitochondrial EngB2, was grouped with the Bacteroides clade, suggesting an LGT origin of type 1 genes from Bacteroides. On the other hand, the type 2 cluster, containing chloroplast-targeting EngB3, was grouped with a cluster from α-proteobacteria. Fungi and protist genes were closely related to this clade, but animal genes formed a distinct cluster (100%) that was related to the archaeal cluster, suggesting that type 2 genes were derived from α-proteobacteria. It is expected that α-proteobacteria-related fungal and protist EngB GTPases are localized to mitochondria. Animals probably have lost the type 2 EngB genes although fungi, protists and plants retain them. Type 1 EngB was conserved in *C. merolae*, but the type 2 EngB was not. These results suggest that the mitochondrion-derived EngB3 has changed its target from mitochondria to chloroplasts.

### Archaea-related Drg and Nog1 target to the cytoplasm and nucleus, respectively

Eubacteria possess two Obg family proteins, Obg and EngD, which are also conserved in plants and animals. By contrast, archaea encode two other Obg-related proteins, Drg and Nog1. In addition to eubacterium-like Obg and EngD GTPases, all eukaryotes possess Drg and Nog1, suggesting their distinct roles in eukaryotic cells. It has been shown that Drg GTPases are associated with translating ribosomes in the cytoplasm in *S. cerevisiae* (Li and Trueb, 2000). On the other hand, NOG1 is critical for biogenesis of the 60S ribosomal subunit in the nucleus (Jensen et al., 2003). Arabidopsis encodes three Drg (Drg1–Drg3) and two Nog1 (Nog1-1, Nog1-2) homologs. Subcellular localization analyses using GFP fusion proteins revealed that all Drg GTPases are localized to the cytoplasm in Arabidopsis (Suwastika et al., 2014), whereas NOG1 homologs were localized to the nucleus (Figure 4). Phylogenetic analyses of Drg and Nog1 proteins revealed that both Drg and Nog1 proteins formed a distinct monophyletic cluster with 97% and 100% support, respectively (Figures S8, S9). Plant Drg and Nog1 were related to archaeal Drg and Nog1 proteins. These results suggest that Obg-related Drg and Nog1 GTPases were derived from archaeal GTPases and have acquired specific functions in the cytoplasm and nucleus, respectively, during evolution.

## Discussion

Chloroplasts are descended from an ancient endosymbiotic cyanobacterium. Consequently, it has been thought that nuclear genes encoding chloroplast proteins are mainly derived from the endosymbiotic cyanobacterium. Indeed, it is estimated that 14–18% of nuclear-encoded proteins are cyanobacterial in origin (Martin et al., 2002; Deusch et al., 2008). However, chloroplast proteins are not only encoded by cyanobacterium-derived genes, but also by a considerable number of non-cyanobacterial genes. Chloroplasts have recruited significant number of eukaryotic proteins from host cells. Thus, chloroplasts possess unique prokaryotic-eukaryotic hybrid systems in several cellular processes, including transcription (Baumgartner et al., 1993; Yagi and Shiina, 2011), translation and metabolic pathways (Martin and Schnarrenberger, 1997; Reyes-Prieto and Bhattacharya, 2007; Reyes-Prieto and Moustafa, 2012). In total, more than 600 non-cyanobacterial-host-derived proteins contribute to the chloroplast proteome, which includes ~3000 proteins (Abdallah et al., 2000). It has been suggested that Chlamydia genomes encode a large number of plant-related genes (Brinkman et al., 2002). Moreover, previous study identified 31 genes highly related to those from Chlamydiae in green algae and plants, and 20 Chlamydiae-related genes shared by red and green algae (Moustafa et al., 2008). Another study identified 39 proteins of chlamydial origin in photosynthetic eukaryotes (Becker et al., 2008). Chlamydiae are obligate intracellular pathogens/symbionts in many eukaryotes, although not in plants. It is presumed that Chlamydiae temporarily established an endosymbiosis with ancestral plant cells containing chloroplasts and transferred a number of genes into the host cell (Becker et al., 2008; Moustafa et al., 2008). Some evidence suggests that the LGT of chlamydial genes occurred before the divergence of the Glaucoplantae, Rhodoplantae and Viridiplantae (Becker et al., 2008). In addition, several lines of evidence suggest that there were LGTs among other eubacteria and plants. It has been reported that the gene for chloroplast-localized rRNA adenine dimethyltransferase (rAD) was acquired by LGT from Bacteroides/chlorobi in the rhodophyte lineages, whereas rAD genes of chlorophytes/land plants are derived from Chlamydiae genes (Park et al., 2009). Genes for plastid-localized shikimate pathway proteins are derived from prokaryotic sources, including a proteobacterium related to the γ/β group and an α-proteobacterium (Waller et al., 2006). Furthermore, it has been suggested that some enzymes encoded in the host nuclear genome were mistargeted into the plastid during the evolution of plastids (Reyes-Prieto and Moustafa, 2012). In this study, we found that three chloroplast GTPases (EngD2, EngA2, and EngB3) are likely derived from α-proteobacterium-like ancestors, suggesting re-compartmentation of mitochondrial GTPases.

**Figure 9:**
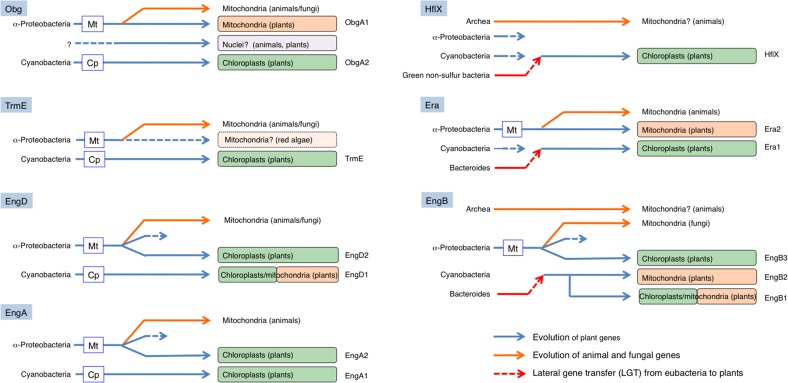
**Schematic model of the evolution of eubacteria-type plant Era-like genes**. Chloroplasts in land plants likely have acquired four GTPases from cyanobacteria by endosymbiotic gene transfer (EGT): ObgA2, TrmE, EngD1 (dual targeting), and EngA1. In addition, mitochondria have also acquired two GTPases from α-proteobacteria through EGT; ObgA1, and Era2. On the other hand, chloroplasts have acquired three α-proteobacterium-related GTPases, probably through re-compartmentation of mitochondrial proteins: EngD2, EngA2, and EngB3. Moreover, chloroplasts have acquired one GTPase from Green non-sulfur bacteria (HflX) and two GTPases from Bacteroides [Era1 and EngB-2 (dual-targeting)]. Similarly, mitochondria also have acquired one GTPase from Bacteroides (EngB2).

We also identified two novel LGT events among eubacteria and plants. Figure 9 shows a summary of possible evolutionary models including LGT for the Era-like GTPase subfamily genes. First, we found that three genes (chloroplast-targeting Era1, chloroplast/mitochondrion dual-targeting EngB1 and mitochondrion-targeting EngB2) were acquired from Bacteroides through LGT. In these cases, cyanobacterium-derived homologs were likely replaced by novel genes and have disappeared during evolution. In contrast to the situation for rAD, Bacteroides-related Era1, EngB1, and EngB2 genes were also found in red algae *C. merolae*, suggesting that the LGT event occurred before the divergence of the Glaucoplantae, Rhodoplantae, and Viridiplantae. Secondly, we found that LGT from green non-sulfur bacteria to plants provided a novel type of chloroplast-localized HflX in plants. This is the first evidence of LGT from non-oxygen producing photosynthetic eubacteria to plants. It remains unclear whether green non-sulfur bacterium-derived HflX confers any functional advantage in chloroplasts compared to the cyanobacterium-related gene. Taken together, our work demonstrates that LGT from eubacteria to plants occurred more frequently than previously thought. It is plausible that eubacterial genes provided novel functions in chloroplasts and that they played a crucial role in plant evolution.

### Conflict of interest statement

The authors declare that the research was conducted in the absence of any commercial or financial relationships that could be construed as a potential conflict of interest.

## Supplementary material

Figure S1 **Phylogenetic tree of Obg subfamily proteins**. Comprehensive comparison of Obg subfamily proteins in eukaryotes, eubacteria and archaea. Sequences were aligned using Clustal X based on 185 proteins. The tree was inferred using the neighbor-joining method with JTT model. Numbers at the nodes indicate bootstrap values obtained for 100 replicates.

Figure S2 **Phylogenetic tree of TrmE subfamily proteins**. Comprehensive comparison of TrmE subfamily proteins in eukaryotes, eubacteria and archaea. Sequences were aligned using Clustal X based on 152 proteins. The tree was inferred using the neighbor-joining method with JTT model. Numbers at the nodes indicate bootstrap values obtained for 100 replicates.

Figure S3 **Phylogenetic tree of EngD subfamily proteins**. Comprehensive comparison of EngD subfamily proteins in eukaryotes, eubacteria and archaea. Sequences were aligned using Clustal X based on 182 proteins. The tree was inferred using the neighbor-joining method with JTT model. Numbers at the nodes indicate bootstrap values obtained for 100 replicates.

Figure S4 **Phylogenetic tree of EngA subfamily proteins**. Comprehensive comparison of EngA subfamily proteins in eukaryotes, eubacteria and archaea. Sequences were aligned using Clustal X based on 147 proteins. The tree was inferred using the neighbor-joining method with JTT model. Numbers at the nodes indicate bootstrap values obtained for 100 replicates.

Figure S5 **Phylogenetic tree of HflX subfamily proteins**. Comprehensive comparison of HflX subfamily proteins in eukaryotes, eubacteria and archaea. Sequences were aligned using Clustal X based on 153 genes. The tree was inferred using the neighbor-joining method with JTT model. Numbers at the nodes indicate bootstrap values obtained for 100 replicates.

Figure S6 **Phylogenetic tree of Era subfamily proteins**. Comprehensive comparison of Era subfamily proteins in eukaryotes, eubacteria and archaea. Sequences were aligned using Clustal X based on 141 proteins. The tree was inferred using the neighbor-joining method with JTT model. Numbers at the nodes indicate bootstrap values obtained for 100 replicates.

Figure S7 **Phylogenetic tree of EngB subfamily proteins**. Comprehensive comparison of EngB subfamily proteins in eukaryotes, eubacteria and archaea. Sequences were aligned using Clustal X based on 143 proteins. The tree was inferred using the neighbor-joining method with JTT model. Numbers at the nodes indicate bootstrap values obtained for 100 replicates.

Figure S8 **Phylogenetic tree of Drg subfamily proteins**. Comprehensive comparison of Drg subfamily proteins in eukaryotes, eubacteria and archaea. Sequences were aligned using Clustal X based on 185 proteins. The tree was inferred using the neighbor-joining method with JTT model. Numbers at the nodes indicate bootstrap values obtained for 100 replicates.

Figure S9 **Phylogenetic tree of Nog subfamily proteins**. Comprehensive comparison of Nog1 subfamily proteins in eukaryotes, eubacteria and archaea. Sequences were aligned using Clustal X based on 185 proteins. The tree was inferred using the neighbor-joining method with JTT model. Numbers at the nodes indicate bootstrap values obtained for 100 replicates.
