# Supplementary figures and images for: Evidence for lateral gene transfer (LGT) in the evolution of eubacteria-derived small GTPases in plant organelles
Source: Front Plant Sci. 2014 Dec 11;5:678. doi: 10.3389/fpls.2014.00678 (PMC4263083; doi:10.3389/fpls.2014.00678)

Figure S1

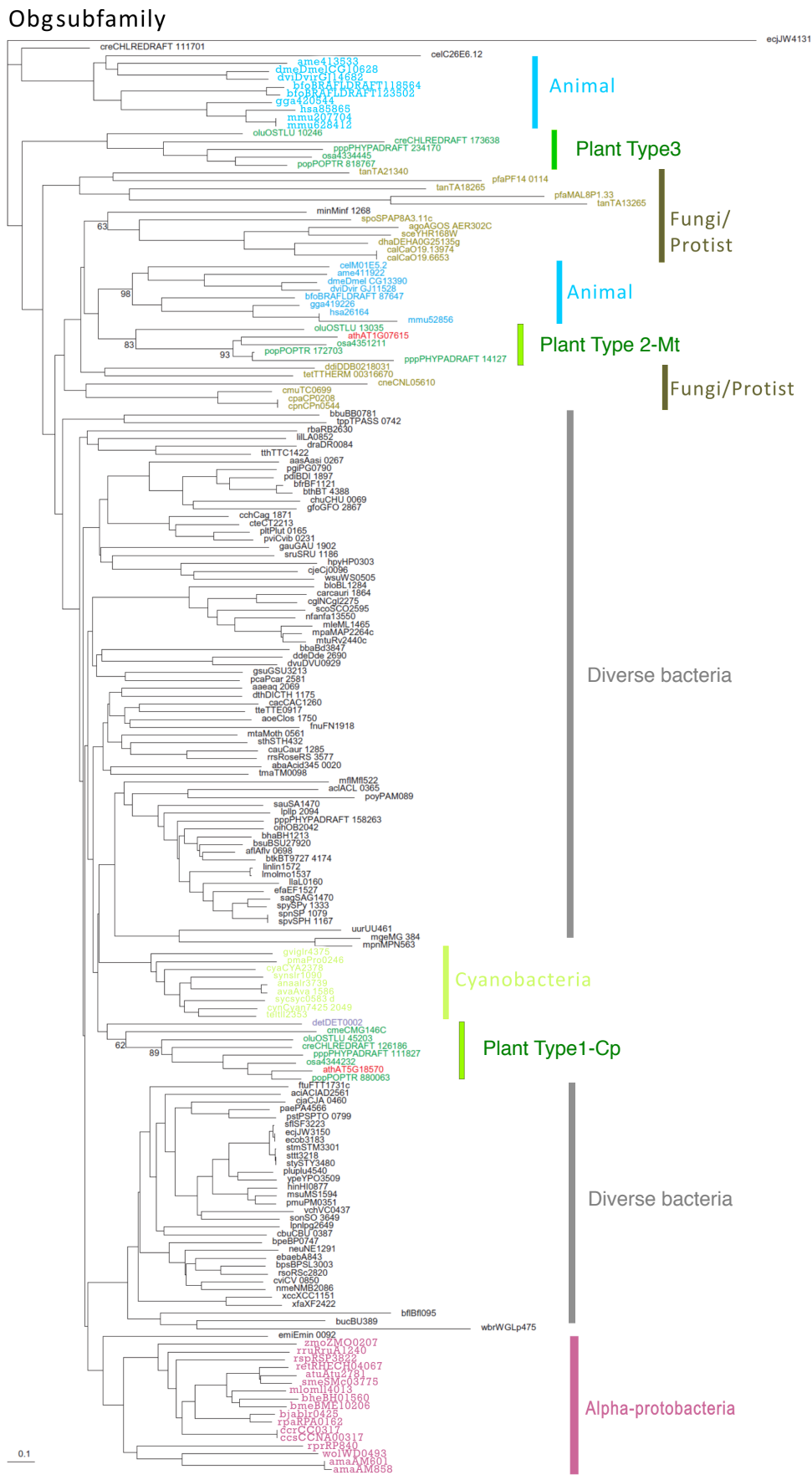

Supplement: Figure S1 — Phylogenetic tree of Obg subfamily proteins. Comprehensive comparison of Obg subfamily proteins in eukaryotes, eubacteria and archaea. Sequences were aligned using Clustal X based on 185 proteins. The tree was inferred using the neighbor-joining method with JTT model. Numbers at the nodes indicate bootstrap values obtained for 100 replicates. [file Image1.PDF]

Figure S2

TrmE family

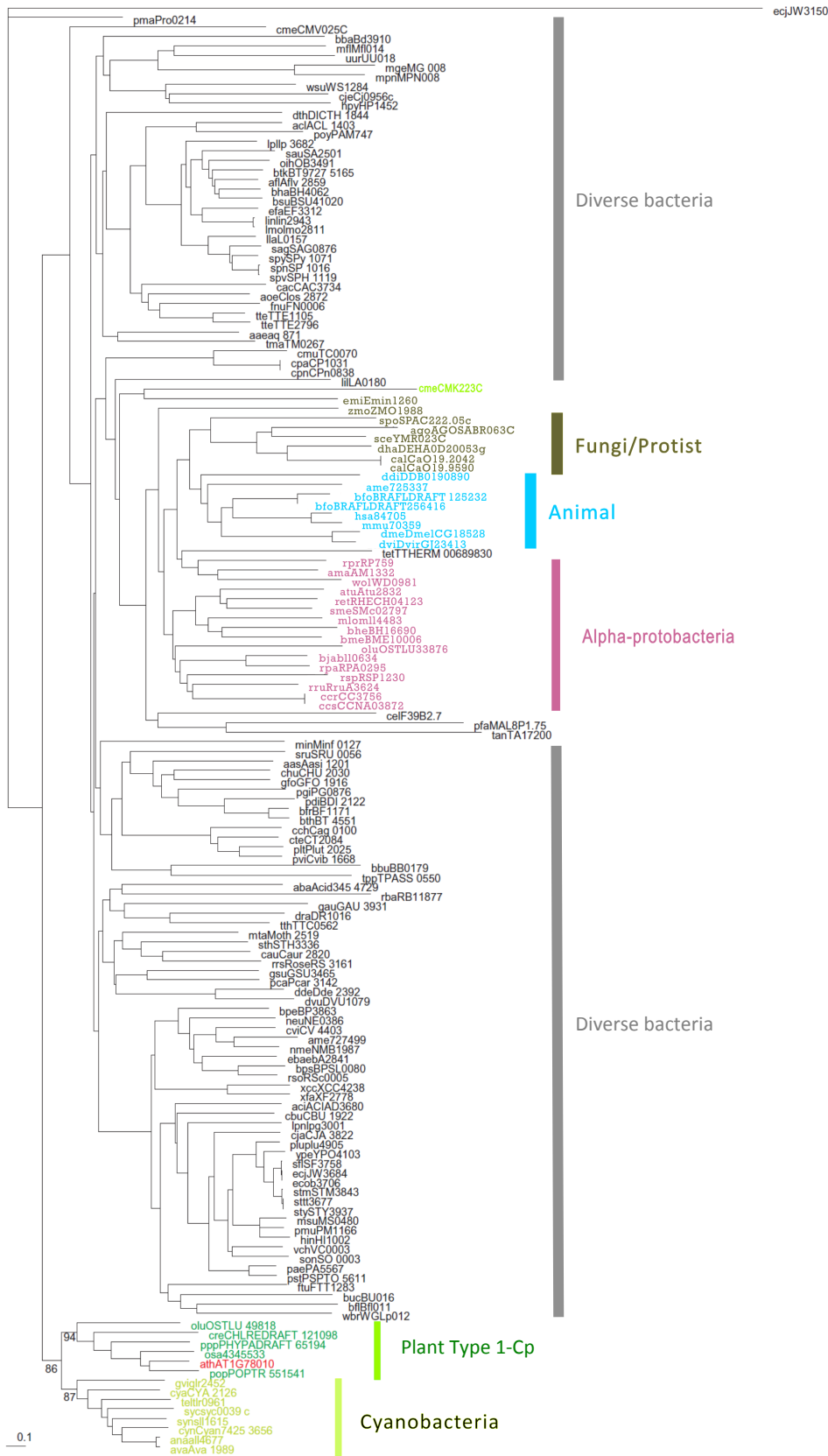

Supplement: Figure S2 — Phylogenetic tree of TrmE subfamily proteins. Comprehensive comparison of TrmE subfamily proteins in eukaryotes, eubacteria and archaea. Sequences were aligned using Clustal X based on 152 proteins. The tree was inferred using the neighbor-joining method with JTT model. Numbers at the nodes indicate bootstrap values obtained for 100 replicates. [file Image2.PDF]

Figure S3

EngD subfamily

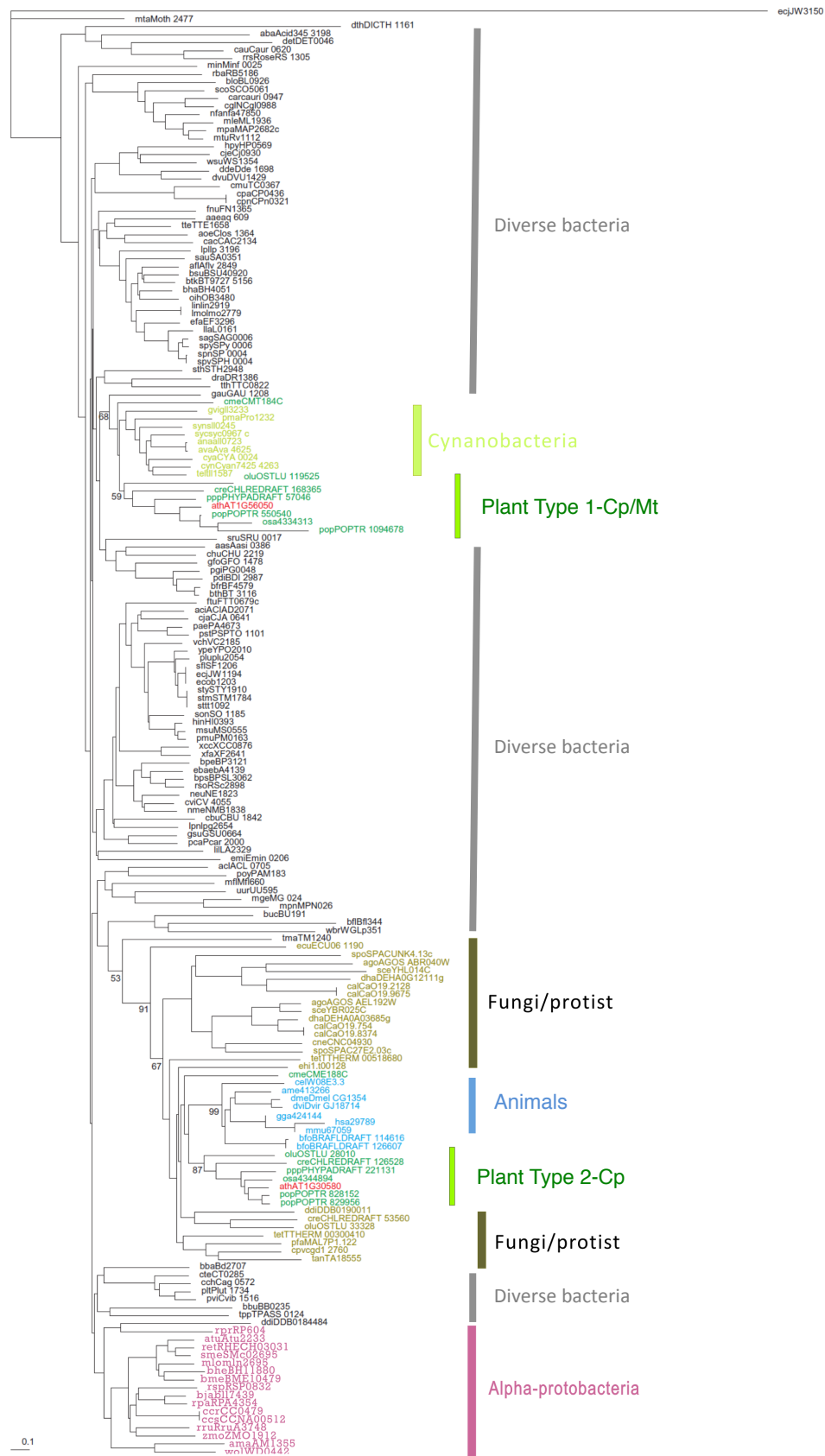

Supplement: Figure S3 — Phylogenetic tree of EngD subfamily proteins. Comprehensive comparison of EngD subfamily proteins in eukaryotes, eubacteria and archaea. Sequences were aligned using Clustal X based on 182 proteins. The tree was inferred using the neighbor-joining method with JTT model. Numbers at the nodes indicate bootstrap values obtained for 100 replicates. [file Image3.PDF]

Figure S4

EngA family

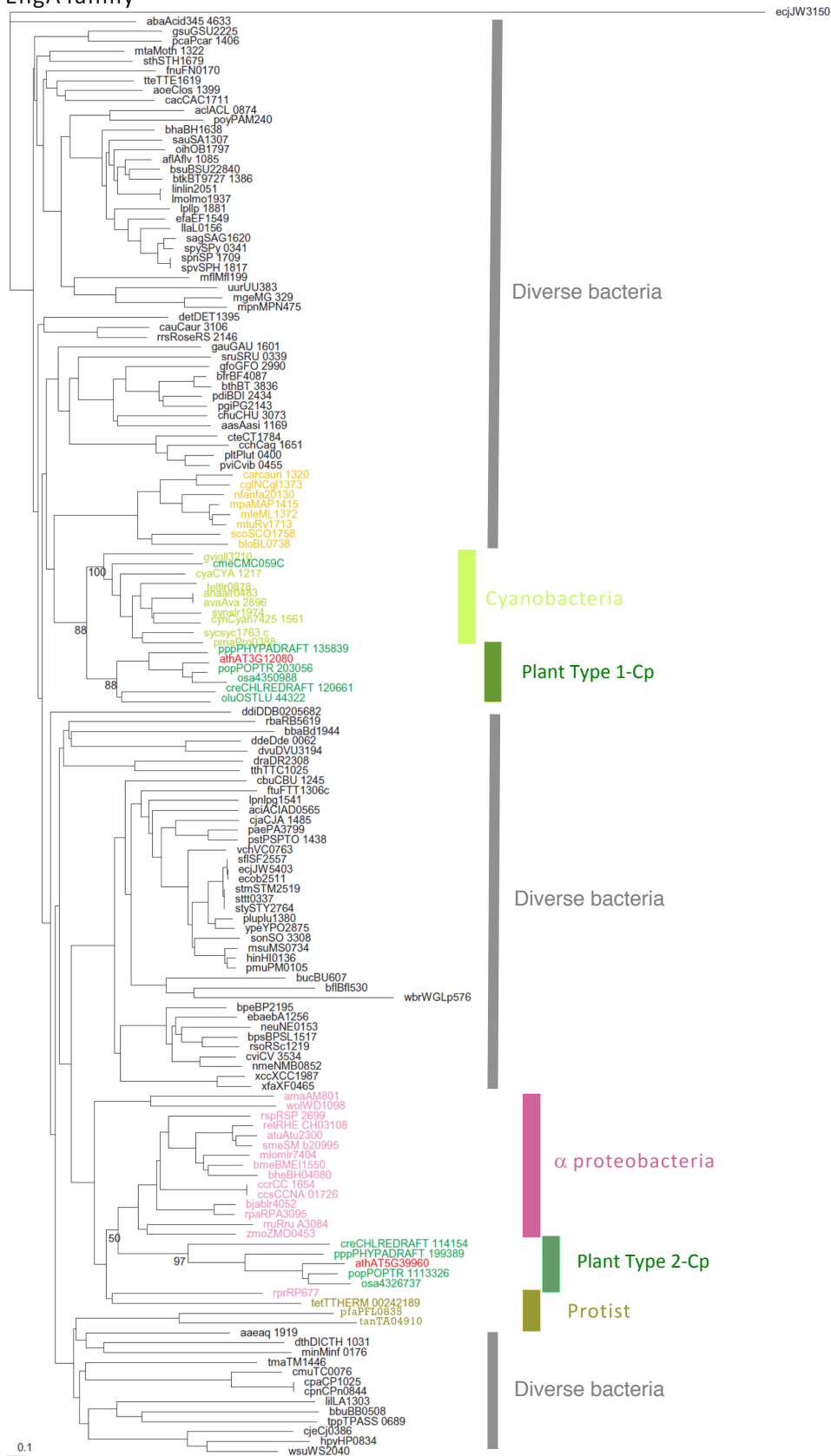

Supplement: Figure S4 — Phylogenetic tree of EngA subfamily proteins. Comprehensive comparison of EngA subfamily proteins in eukaryotes, eubacteria and archaea. Sequences were aligned using Clustal X based on 147 proteins. The tree was inferred using the neighbor-joining method with JTT model. Numbers at the nodes indicate bootstrap values obtained for 100 replicates. [file Image4.PDF]

Figure S5

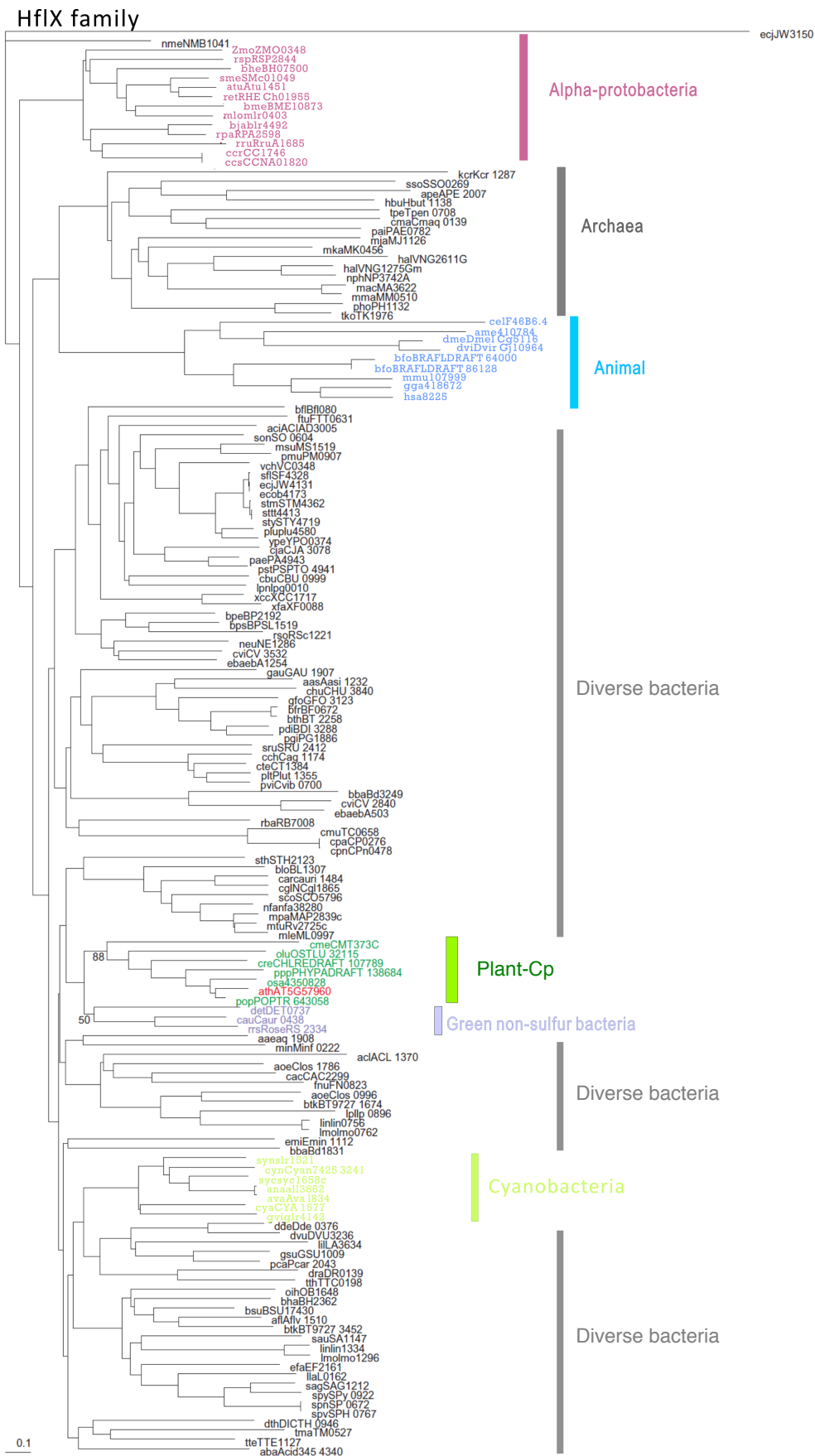

Supplement: Figure S5 — Phylogenetic tree of HflX subfamily proteins. Comprehensive comparison of HflX subfamily proteins in eukaryotes, eubacteria and archaea. Sequences were aligned using Clustal X based on 153 genes. The tree was inferred using the neighbor-joining method with JTT model. Numbers at the nodes indicate bootstrap values obtained for 100 replicates. [file Image5.PDF]

Figure S6

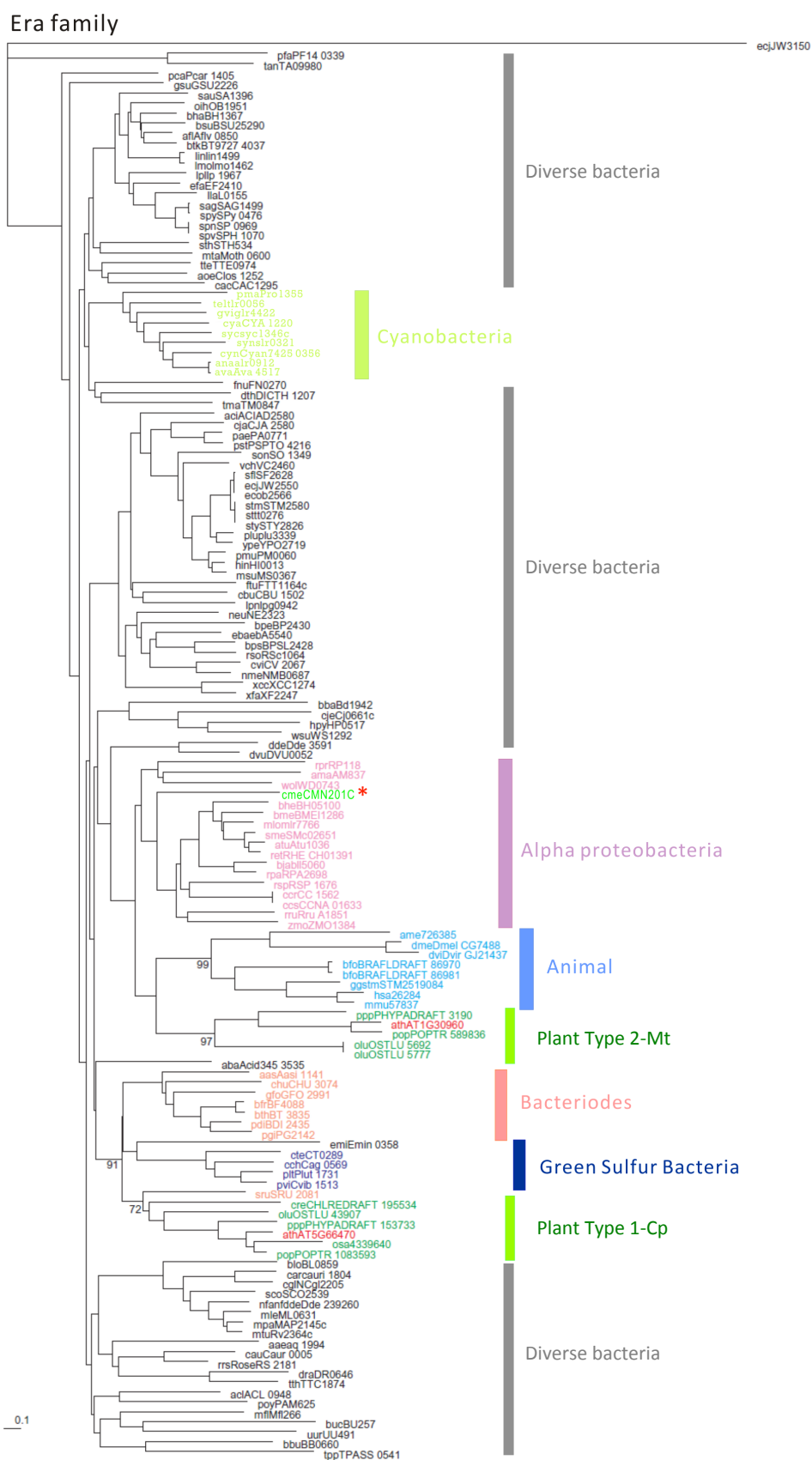

Supplement: Figure S6 — Phylogenetic tree of Era subfamily proteins. Comprehensive comparison of Era subfamily proteins in eukaryotes, eubacteria and archaea. Sequences were aligned using Clustal X based on 141 proteins. The tree was inferred using the neighbor-joining method with JTT model. Numbers at the nodes indicate bootstrap values obtained for 100 replicates. [file Image6.PDF]

Figure S7

EngB family

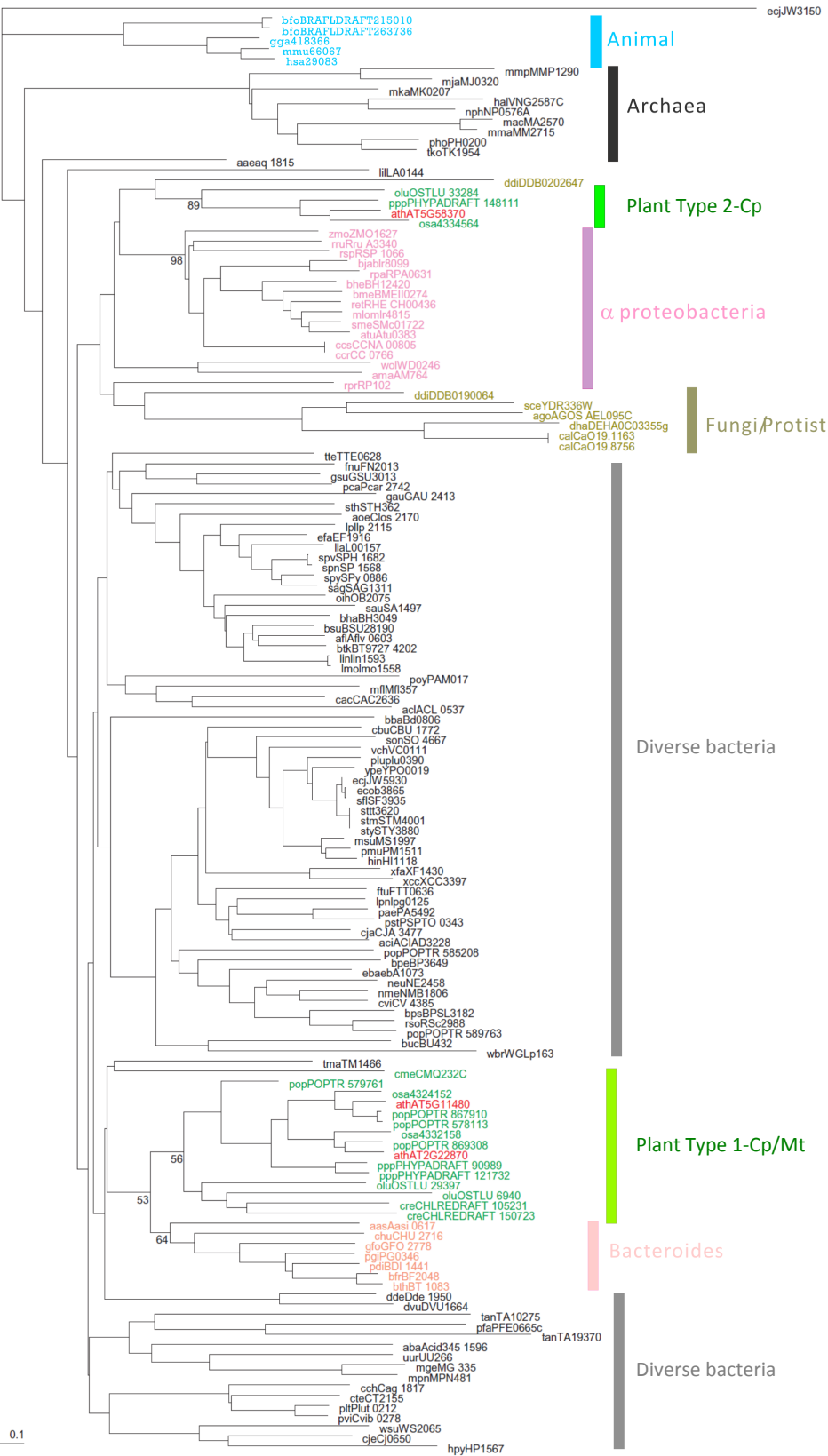

Supplement: Figure S7 — Phylogenetic tree of EngB subfamily proteins. Comprehensive comparison of EngB subfamily proteins in eukaryotes, eubacteria and archaea. Sequences were aligned using Clustal X based on 143 proteins. The tree was inferred using the neighbor-joining method with JTT model. Numbers at the nodes indicate bootstrap values obtained for 100 replicates. [file Image7.PDF]

Figure S8

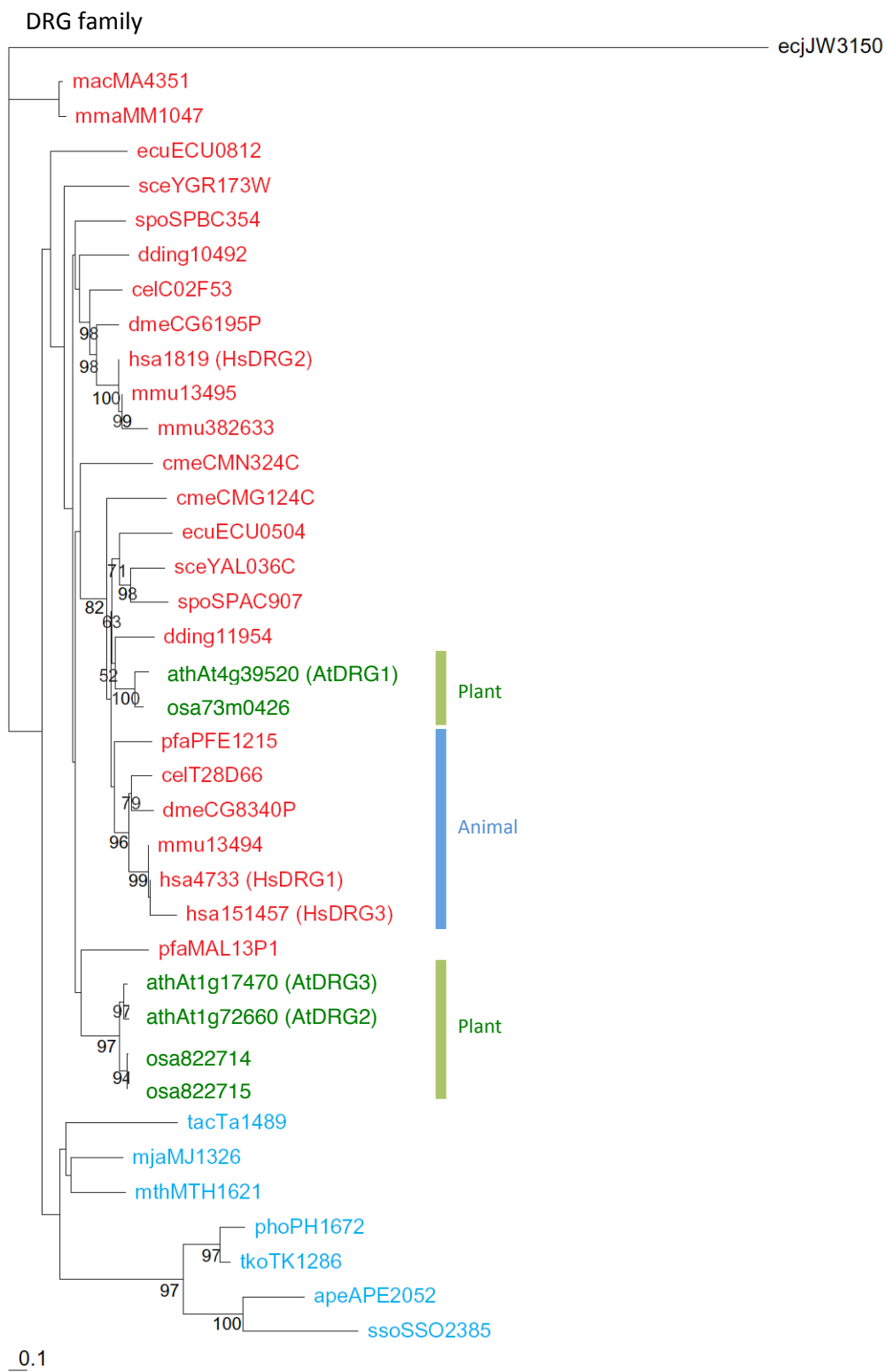

Supplement: Figure S8 — Phylogenetic tree of Drg subfamily proteins. Comprehensive comparison of Drg subfamily proteins in eukaryotes, eubacteria and archaea. Sequences were aligned using Clustal X based on 185 proteins. The tree was inferred using the neighbor-joining method with JTT model. Numbers at the nodes indicate bootstrap values obtained for 100 replicates. [file Image8.PDF]

Figure S9

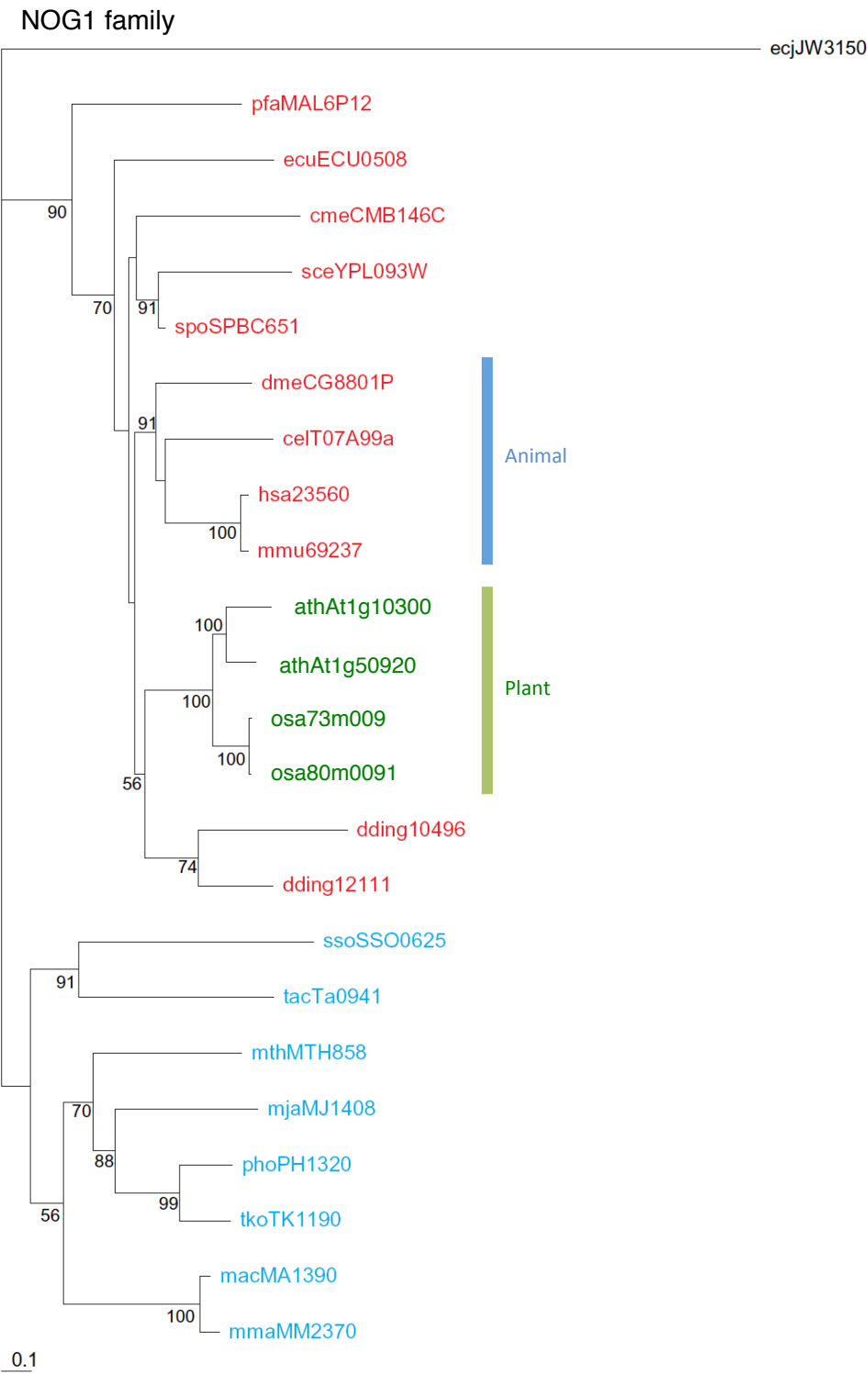

Supplement: Figure S9 — Phylogenetic tree of Nog subfamily proteins. Comprehensive comparison of Nog1 subfamily proteins in eukaryotes, eubacteria and archaea. Sequences were aligned using Clustal X based on 185 proteins. The tree was inferred using the neighbor-joining method with JTT model. Numbers at the nodes indicate bootstrap values obtained for 100 replicates. [file Image9.PDF]
